# Ethylene signals modulate the survival of *Arabidopsis* leaf explants

**DOI:** 10.1186/s12870-023-04299-4

**Published:** 2023-05-26

**Authors:** Seung Yong Shin, Chae-Min Lee, Hyun-Soon Kim, Changsoo Kim, Jae-Heung Jeon, Hyo-Jun Lee

**Affiliations:** 1 Plant Systems Engineering Research Center, Korea Research Institute of Bioscience and Biotechnology, Daejeon, 34141 Korea; 2 Department of Functional Genomics, KRIBB School of Bioscience, University of Science and Technology, Daejeon, 34113 Korea; 3 Department of Crop Science, Chungnam National University, Daejeon, 34134 Korea; 4 Department of Biosystems and Bioengineering, KRIBB School of Biotechnology, University of Science and Technology, Daejeon, 34113 Korea; 5 Department of Biological Sciences, Sungkyunkwan University, Suwon, 16419 Korea

**Keywords:** Ethylene, Survival of explants, Defense gene expression, Anthocyanin, Arabidopsis

## Abstract

**Background:**

Leaf explants are major materials in plant tissue cultures. Incubation of detached leaves on phytohormone-containing media, which is an important process for producing calli and regenerating plants, change their cell fate. Although hormone signaling pathways related to cell fate transition have been widely studied, other molecular and physiological events occurring in leaf explants during this process remain largely unexplored.

**Results:**

Here, we identified that ethylene signals modulate expression of pathogen resistance genes and anthocyanin accumulation in leaf explants, affecting their survival during culture. Anthocyanins accumulated in leaf explants, but were not observed near the wound site. Ethylene signaling mutant analysis revealed that ethylene signals are active and block anthocyanin accumulation in the wound site. Moreover, expression of defense-related genes increased, particularly near the wound site, implying that ethylene induces defense responses possibly by blocking pathogenesis via wounding. We also found that anthocyanin accumulation in non-wounded regions is required for drought resistance in leaf explants.

**Conclusions:**

Our study revealed the key roles of ethylene in the regulation of defense gene expression and anthocyanin biosynthesis in leaf explants. Our results suggest a survival strategy of detached leaves, which can be applied to improve the longevity of explants during tissue culture.

**Supplementary Information:**

The online version contains supplementary material available at 10.1186/s12870-023-04299-4.

## Background

Plant tissue culture is an important technique to produce calli and regenerated plants, which are used as materials in various industries. For example, regenerated *Sophora flavescens* Aiton and *Rhodiola imbricata* contain higher levels of medicinal phytochemicals, including maackiain and cinnamyl alcohol, than the normally grown plants [1, 2], suggesting the importance of plant regeneration in the food and pharmaceutical industries. In addition, *Bougainvillea glabra* and *Artemisia annua* L. calli produce flavonoids and phenolic acids as antioxidants [3, 4]. To induce callus formation or shoot/root organogenesis, plant tissues are excised and incubated on a medium containing various phytohormones. Low levels of auxins induce adventitious root organogenesis, whereas high levels of auxins promote cell dedifferentiation to induce callus formation [5–8]. In contrast, a high cytokinin:auxin ratio changes the cell fate and promotes the formation of shoot meristems [9]. In these processes, the explant conditions are important for efficient tissue culture. In *Arabidopsis thaliana* (Arabidopsis), the efficiency of root organogenesis in leaf explants is highly dependent on the leaf age [5]. In addition, salinity stress in leaf explants largely decreases the rates of callus formation and shoot regeneration in tomatoes [10]. However, molecular and physiological events other than those affecting callus formation and organogenesis in leaf explants remain largely elusive.

Ethylene is a gaseous hormone that regulates various physiological responses in plants. Ethylene biosynthesis is mediated by 1-aminocyclopropane-1-carboxylic acid synthases (ACSs) [11]. In Arabidopsis, the expression of *ACS2*, *ACS6*, *ACS7*, and *ACS8* is up-regulated by mitogen-activated and calcium-dependent protein kinases after wounding or infection by pathogens, such as *Botrytis cinerea* and *Pseudomonas syringae* pv. *tomato* DC3000 [12–14]. Ethylene activates downstream signaling pathways via endoplasmic reticulum-localized ethylene receptors and nearby signaling proteins [15]. ETHYLENE INSENSITIVE 2 (EIN2) acts as a signaling hub [16, 17]. Mutations in *EIN2* cause defects in ethylene responses, including resistance to *B. cinerea* [18, 19]. EIN2 up-regulates the *ETHYLENE RESPONSE FACTOR 1* (*ERF1*) expression in *B. cinerea*-infected plants [20, 21]. ERF1 induces the expression of defense-related genes, including *PLANT DEFENSIN 1.2* (*PDF1.2*) and *PATHOGENESIS-RELATED 3* (*PR3*), to confer resistance to *B. cinerea*. EIN2 is also involved in resistance to *Fusarium* [19, 22]. The *ein2* mutant plants exhibit defects to induce the expression of *ERF1* and *PDF1.2* after infection with *Fusarium graminearum* and *F. oxysporum* [19], indicating that EIN2 plays an important role in pathogen resistance responses.

Anthocyanins are phytochemicals that accumulate in various tissues of plants under abiotic stress conditions [23]. Accumulation of anthocyanins protects the plants from stress-induced damage, possibly via their roles as antioxidants [24]. In addition to pathogen resistance response, ethylene also plays a key role in abiotic stress-induced anthocyanin biosynthesis [25–27]. Inhibition of ethylene signaling by ethylene signaling inhibitors increases sugar-mediated anthocyanin accumulation in plants [25]. In contrast, the *CONSTITUTIVE TRIPLE RESPONSE1* (*CTR1*)-deficient mutant that exhibits constitutive ethylene responses shows reduced anthocyanin accumulation [25]. In addition, mutations in ethylene signaling transcription factors, *ETHYLENE-INSENSITIVE 3* (*EIN3*) and *EIN3-LIKE 1* (*EIL1*), increase anthocyanin levels under high sucrose conditions [27]. Moreover, ethylene-responsive transcription factors, PpERF9 and PpERF105, inhibit anthocyanin biosynthesis by regulating MYB transcription factors in pear (*Pyrus* spp.) [28, 29]. These results suggest that ethylene negatively regulates anthocyanin accumulation. However, there are opposite results that ethylene promotes anthocyanin biosynthesis in several studies. In Arabidopsis, *ERF4* and *ERF8*-deficient mutants show reduced anthocyanin content and decreased expression of genes involved in anthocyanin biosynthesis under high light stress conditions [30]. In apple (*Malus domestica*), MdEIL1 directly activates *MdMYB1* to induce anthocyanin accumulation [31]. These studies show the importance of ethylene signals in controlling anthocyanin content, yet the regulatory mechanisms can differ depending on plant species and environmental conditions.

In this study, we identified that ethylene signaling controls the pathogen defense responses and anthocyanin biosynthesis in leaf explants, which would be important for survival of explants during tissue culture. We observed that anthocyanin biosynthesis and pathogen resistance responses are regulated differently depending on the distance from the wound in leaf explants. In addition, accumulated anthocyanins were necessary for drought stress resistance in leaf explants, but they were not involved in *de novo* root organogenesis. Our study shows the role of ethylene in affecting the survival of leaf explants, which would be important to improve longevity of leaf explants during tissue culture.

## Results

### Ethylene inhibits anthocyanin accumulation near the wound site in leaf explants

**Fig. 1:**
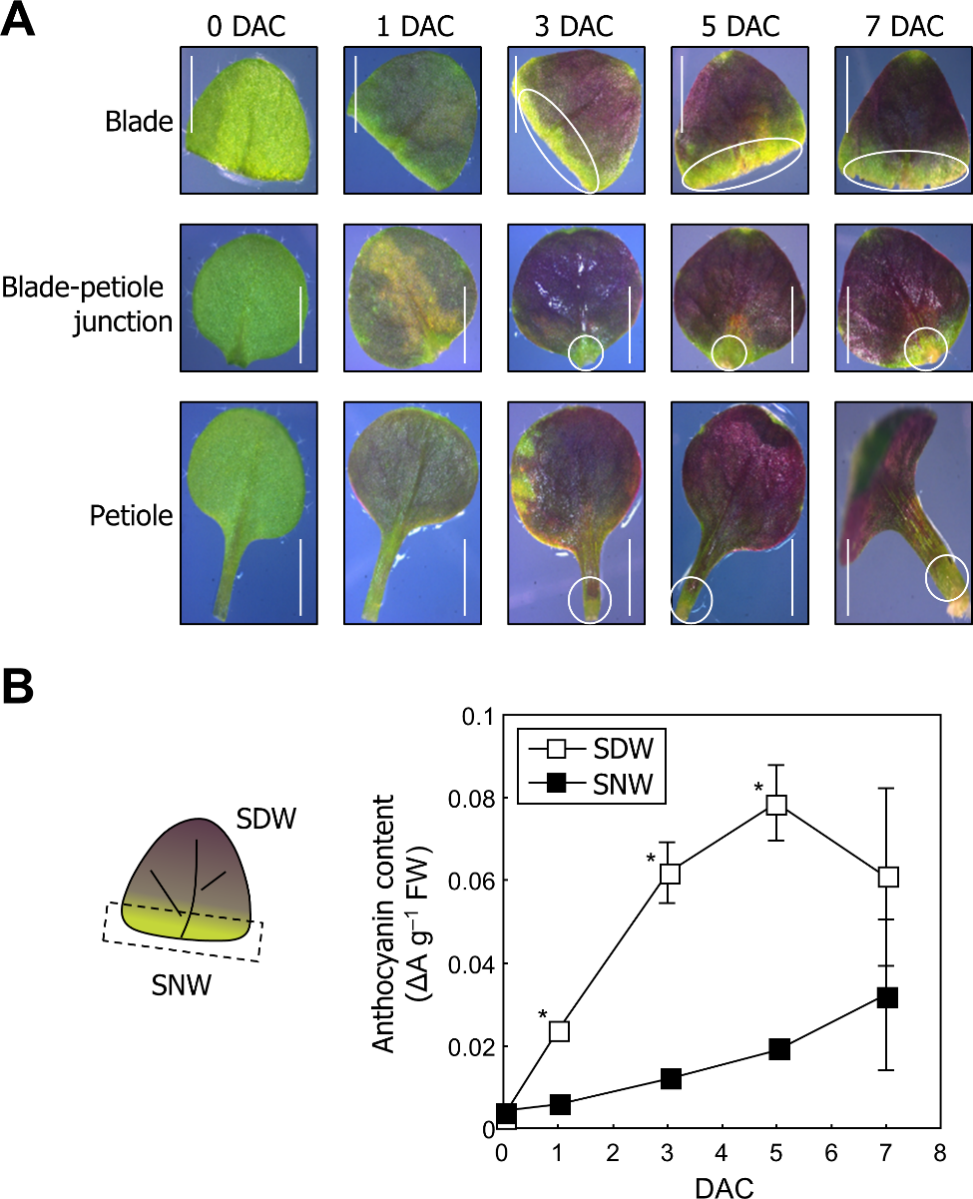
Accumulation of anthocyanins at distant regions from the wound site in leaf explants. (A-B) Col-0 seedlings grown for 9 days were used. (**A**) Phenotype of the leaf explants during tissue culture. Leaves excised at the indicated site (blade, blade-petiole junction, and petiole) were incubated on B5-agar plates for up to 7 days. DAC, days after culture. Size markers indicate 0.25 cm. (**B**) Measurement of anthocyanin content. Leaves excised at blade were used. Anthocyanin contents at the site near the wound (SNW) and at the site distant from the wound (SDW) were separately analyzed. Three biological replicates were averaged and statistically analyzed using Student’s *t*-test (*P < 0.05; difference from SNW). Each replicate contains 5–6 explants. Whiskers indicate ± standard deviations (SD)

We previously reported that wound-induced reactive oxygen species (ROS) and Ca^2+^ are essential for *de novo* root organogenesis in leaf explants [32]. Here, incubation of leaf explants on phytohormone-free medium for *de novo* root organogenesis resulted in the accumulation of anthocyanins (Fig. 1A). Anthocyanin accumulation started one day after culture (DAC) and increased throughout the incubation period. Notably, anthocyanins were not observed at the site near the wound (SNW) until 7 DAC regardless of the excision site (Fig. 1A). To confirm the accumulation patterns of anthocyanins in leaf explants, we separately measured anthocyanin levels at SNW and at a site distant from the wound (SDW). Anthocyanin levels gradually increased at both SNW and SDW, but SDW exhibited significantly higher anthocyanin levels than SNW until 5 DAC (Fig. 1B). These results suggest that anthocyanins accumulated in leaf explants, but wound-induced signals somehow suppress anthocyanin accumulation near the wound site.

**Fig. 2:**
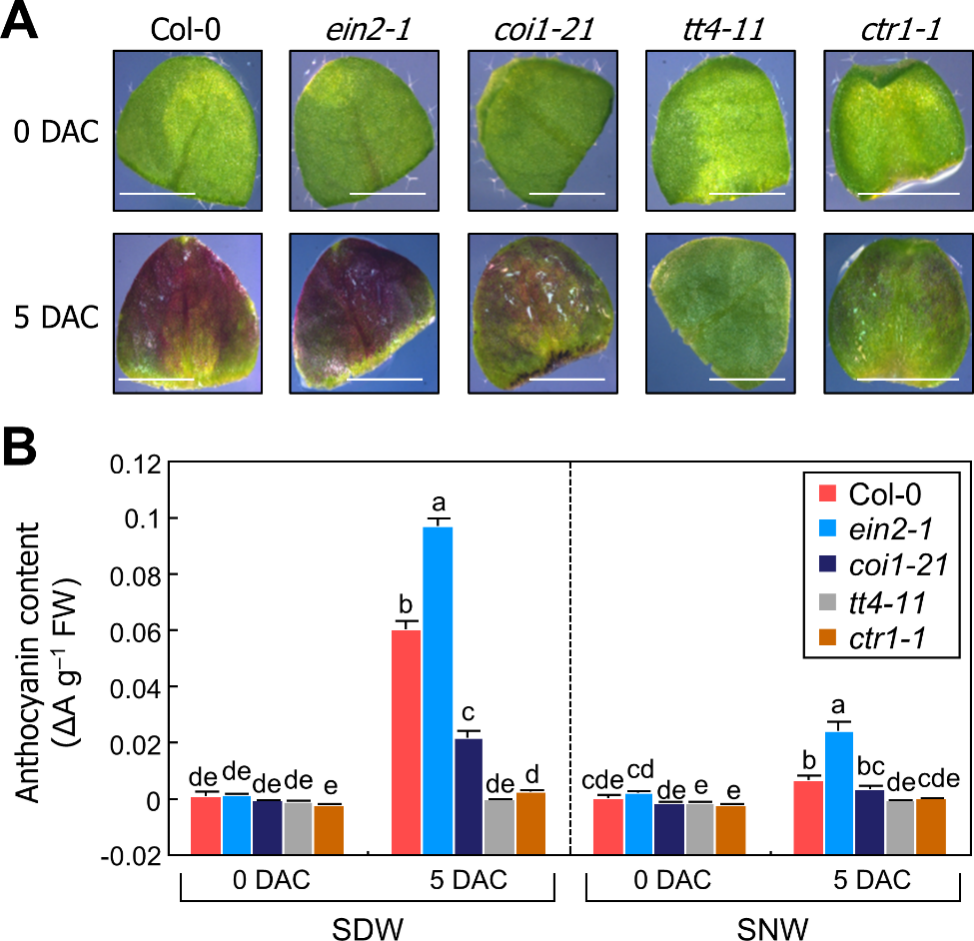
Accumulation of anthocyanins in leaf explants of ethylene signaling mutants. (**A**-**B**) Leaf explants of the 9-day-old Col-0, *ein2-1*, *coi1-21*, *tt4-11*, and *ctr1-1* seedlings were incubated on B5-agar plates for the indicated time periods. (**A**) Representative images are displayed. Size markers indicate 0.125 cm. (**B**) Measurement of anthocyanin content at SDW and SNW. Three biological replicates were averaged. Letters indicate groups that are statistically significantly different from each other (*P* < 0.05, Tukey’s test). Each replicate contains 5–6 explants. Whiskers indicate + SD

As wounding induces jasmonate and ethylene production within 2 h [14, 33], we hypothesized that early phytohormone responses to wounding are related to anthocyanin accumulation patterns in leaf explants. Therefore, we analyzed the anthocyanin levels in mutants defective in ethylene (*ein2-1*) and jasmonate (*coi1-21*) signaling with the *ctr1-1* mutant that exhibits constitutively active ethylene signaling [34–36]. We also used the *tt4-11* mutant that exhibits defective anthocyanin biosynthesis as a control [37]. We found that anthocyanin levels at SDW were significantly reduced by *coi1-21* mutation 5 DAC (Fig. 2A, B). However, the anthocyanin levels at SNW in the *coi1-21* mutant were similar to those in Col-0 wild-type plant, suggesting that only jasmonate signals are required for anthocyanin accumulation at SDW. Moreover, the *ein2-1* mutant showed significantly higher anthocyanin levels than Col-0 at both SDW and SNW. However, the *ctr1-1* mutant showed the opposite trend as its anthocyanin levels were significantly lower than those in Col-0 at both SDW and SNW, similar to *tt4-11*. These results suggest that ethylene signals negatively regulate anthocyanin accumulation in leaf explants, which is consistent with a previous report that ethylene suppresses abiotic stress-induced anthocyanin biosynthesis [25].

### Ethylene signals are activated near the wound site in leaf explants

**Fig. 3:**
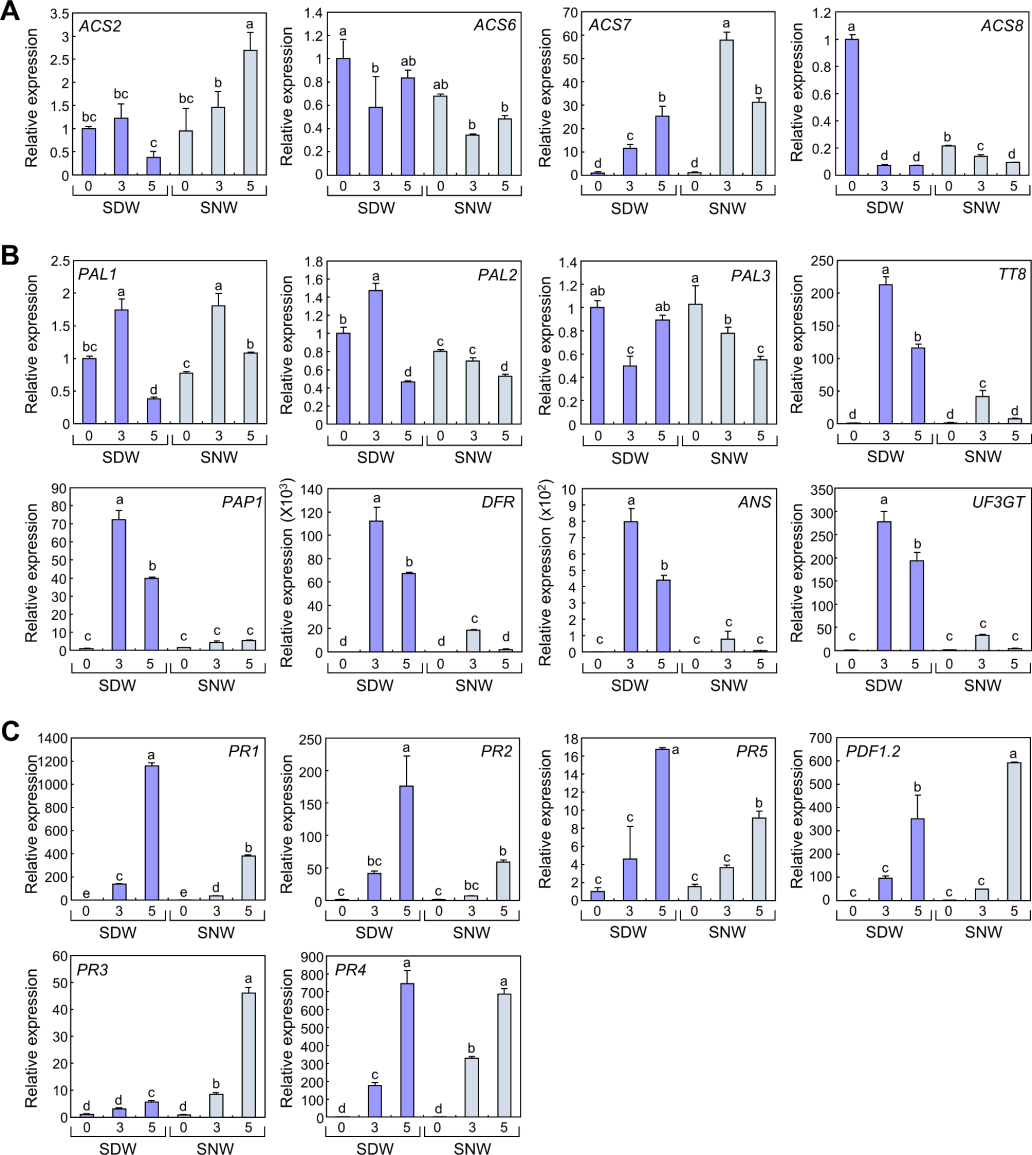
Gene expressions at different regions of the leaf explants. (**A**-**C**) Leaf explants of the 9-day-old Col-0 seedlings were incubated on B5-agar plates for the indicated time periods. Numbers in x-axis indicate DACs. Leaf explants at SDW and SNW were separately harvested. Expression of genes related to ethylene biosynthesis (**A**), anthocyanin biosynthesis (**B**), and defense responses (**C**) was analyzed using RT-qPCR. Technical triplicates were averaged. Letters indicate groups that are statistically significantly different from each other (*P* < 0.05, Tukey’s test). Whiskers indicate + SD

As anthocyanin levels at the SNW were elevated by *ein2-1* mutation (Fig. 2A, B), we hypothesized that ethylene signals are specifically activated near the wound site. To verify whether ethylene production is activated by wounding, we analyzed the expression of representative ethylene biosynthesis genes including *ACS2*, *ACS6*, *ACS7*, and *ACS8*, which are involved in wound-induced ethylene production [14]. Expression of *ACS2* was elevated after leaf excision only at SNW (Fig. 3A). Expression of *ACS7* increased at both SDW and SNW, but expression levels at SNW were significantly higher than those at SDW. Expression of *ACS8* was largely suppressed after leaf excision at SDW, but expression levels became similar at SDW and SNW at 5 DAC. Expression of *ACS6* did not show any significant differences during incubation. These results suggest that ACS2- and ACS7-mediated ethylene biosynthesis might be activated particularly near the wound site during the incubation of leaf explants.

Ethylene has been reported to inhibit anthocyanin biosynthesis by suppressing the transcription of anthocyanin biosynthesis genes [25]. To further analyze the role of ethylene in the spatial regulation of anthocyanin accumulation, we determined the expression of anthocyanin biosynthesis genes. Genes encoding phenylalanine ammonia-lyases (PALs) belong to the phenylpropanoid metabolic pathway, which produces secondary metabolites, including anthocyanins [38]. The PRODUCTION OF ANTHOCYANIN PIGMENT 1 (PAP1) and TRANSPARENT TESTA 8 (TT8) are transcription factors that up-regulate the expression of anthocyanin biosynthesis genes such as *DIHYDROFLAVONOL 4-REDUCTASE* (*DFR*), *ANTHOCYANIDIN SYNTHASE* (*ANS*) and *UDP-GLUCOSE:FLAVONOID 3-O-GLUCOSYLTRANSFERASE (UF3GT)* [39]. Among *PAL* genes, only *PAL2* showed reduced expression at the SNW 0 and 3 DAC, whereas other *PAL*s showed similar and complex expression patterns (Fig. 3B). Notably, expression levels of *TT8* and *PAP1*, along with their downstream target genes *DFR*, *ANS*, and *UF3GT*, were largely increased following leaf excision at SDW. However, the expression levels at SNW were significantly lower than those at SDW. (Fig. 3B). These expression patterns were consistent with low anthocyanin levels and high *ACS*s expression at SNW, suggesting that wound-induced local ethylene production could potentially suppress anthocyanin biosynthesis in the leaf explants.

**Fig. 4:**
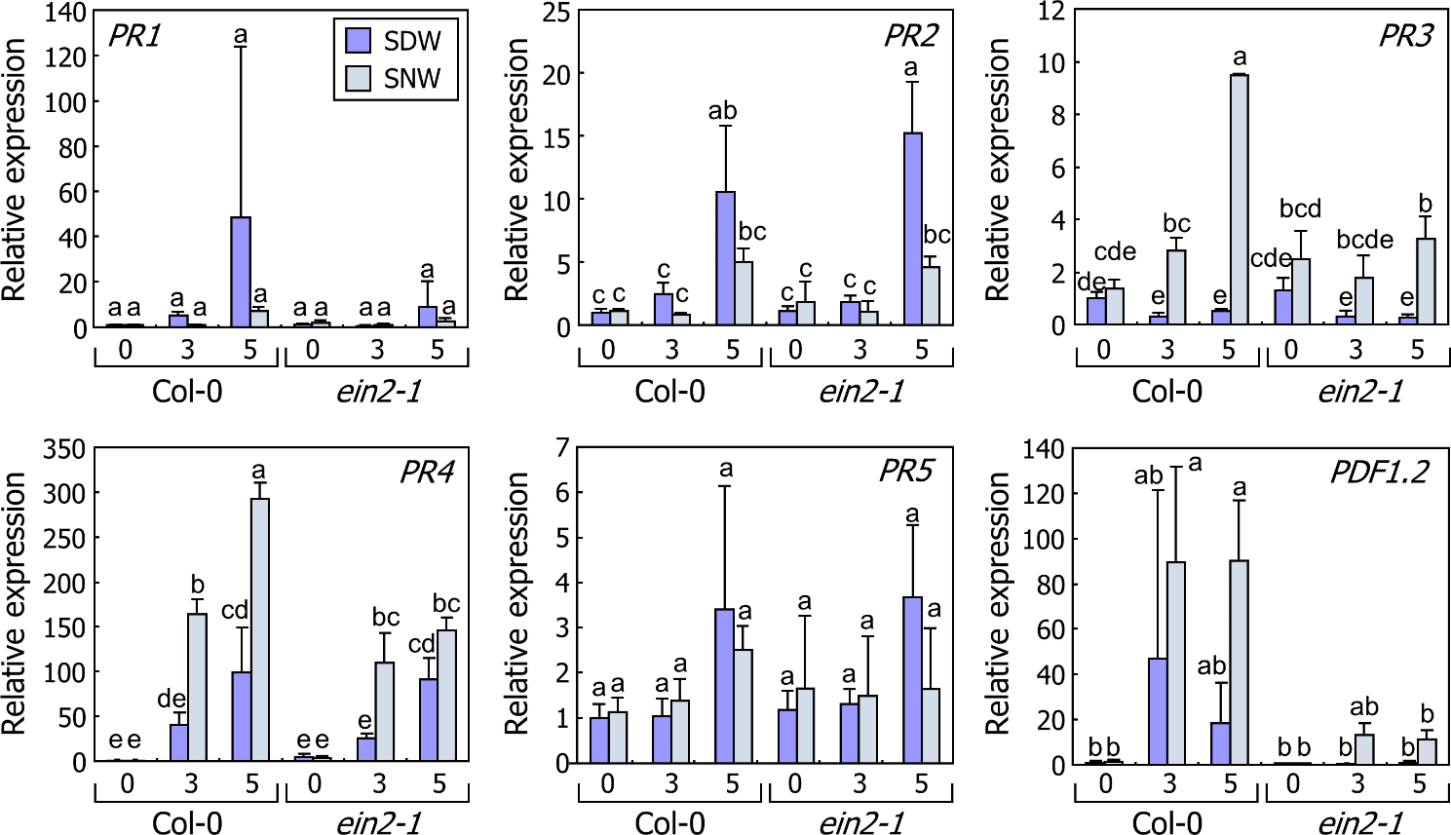
Expression of defense-related genes in leaf explants of the *ein2-1* mutant. Leaf explants of the 9-day-old Col-0 and *ein2-1* seedlings were incubated on B5-agar plates for the indicated time periods. Numbers in x-axis indicate DACs. Leaf explants at SDW and SNW were separately harvested. Biological triplicates were averaged. Letters indicate groups that are statistically significantly different from each other (*P* < 0.05, Tukey’s test). Whiskers indicate + SD

Next, we examined the specific roles of ethylene in leaf explants. Ethylene plays a key role in defense responses to pathogens [18, 19]. Therefore, we hypothesized that ethylene-mediated pathogen resistance is differentially activated at SDW and SNW in leaf explants. To verify our hypothesis, we analyzed the spatial expression patterns of marker genes related to plant defense responses. Expression of all analyzed ethylene-responsive defense genes, *PR3*, *PR4*, and *PDF1.2* [18, 19, 22], was elevated after leaf excision at both SDW and SNW, but the expression levels were significantly higher at SNW than at SDW at least once during incubation (Fig. 3C). In contrast, the expression of salicylic acid marker genes, *PR1*, *PR2*, and *PR5* [40], were significantly higher at SDW than at SNW. To confirm the role of ethylene in the local expression of pathogen resistance genes, we analyzed gene expression in *ein2-1* mutant. Expression of *PR3*, *PR4*, and *PDF1.2* was significantly decreased at SNW, whereas that at SDW was unaltered by *ein2-1* mutation (Fig. 4). In addition, expression of salicylic acid-responsive genes, *PR1* and *PR5*, was not affected by *ein2-1* mutation. Together with our observation that the expression of ethylene biosynthesis genes was elevated at SNW, these results suggest that wound-induced ethylene signals stimulate the defense responses locally near the wound site.

### Roles of anthocyanin in leaf explants

**Fig. 5:**
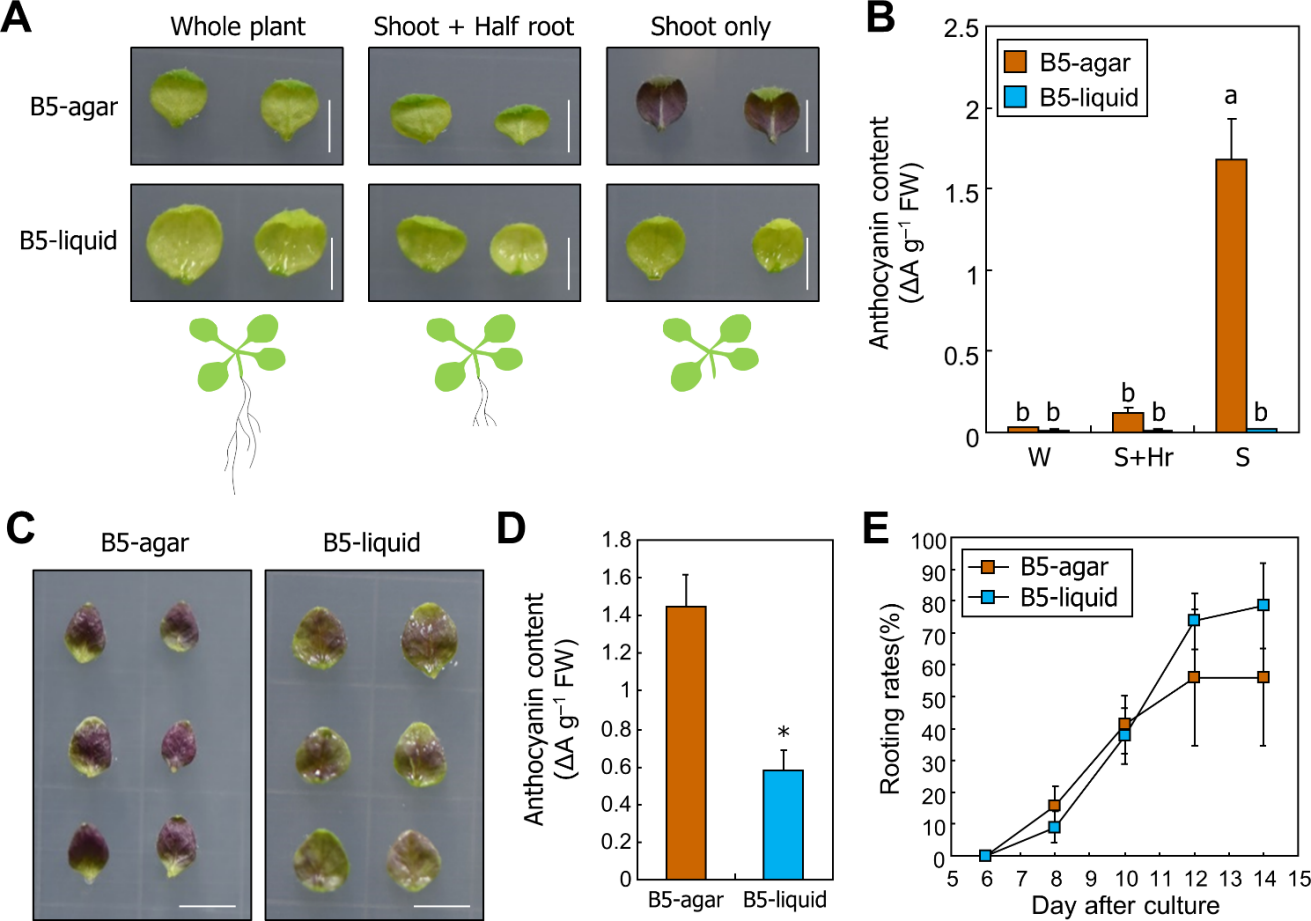
Drought induces anthocyanin biosynthesis in leaf explants. (**A**-**B**) Col-0 seedlings grown for 9 days were used. Whole plant (W), shoot + half root (S + Hr), and shoot only (S) were incubated on B5-agar or -liquid plates for 5 days. (**A**) Representative images are displayed. Size markers indicate 0.5 cm. (**B**) Measurement of anthocyanin content. Three biological replicates were averaged. Letters indicate groups that are statistically significantly different from each other (*P* < 0.05, Tukey’s test). Each replicate contains 5–6 explants. Whiskers indicate + SD. (**C**-**E**) Leaf explants of the 9-day-old Col-0 seedlings were incubated on B5-agar or -liquid plates for 5 days (**C**,**D**) or up to 14 days (**E**). (**C**) Representative images are displayed. Size markers indicate 0.5 cm. (**D**) Anthocyanin content of leaf explants. Six biological replicates were averaged and statistically analyzed using Student’s *t*-test (*P < 0.05; difference from B5-agar). Each replicate contains 5–6 explants. Whiskers indicate + SD. (**E**) Rooting rate of leaf explants incubated on agar and liquid medium. Three biological replicates were averaged and statistically analyzed using Student’s *t*-test (*P < 0.05; difference from B5-agar). Each replicate contains 20–25 explants. Whiskers indicate ± SD

Anthocyanin accumulation is induced by abiotic stresses, such as high light and drought [23, 41, 42]. Accumulated anthocyanins act as a ROS scavengers to protect cells from oxidative damage [23, 41]. As leaf explants do not develop roots until they regenerate, we hypothesized that reduced water intake causes drought stress-induced anthocyanin accumulation. To verify our hypothesis, we incubated aerial parts of the seedlings without entire roots, with half of the roots, and intact seedlings on B5-agar and liquid media. Anthocyanin accumulation was observed only in the seedlings without roots incubated on agar medium (Fig. 5A, B). However, anthocyanin levels were significantly reduced in the seedlings incubated in liquid media, suggesting that decreased water intake through the roots is the major cause for anthocyanin accumulation in the aerial parts of the seedlings.

To investigate whether anthocyanin accumulation in leaf explants is also due to drought stress, we incubated leaf explants on B5-agar and liquid media. As observed in the seedlings, anthocyanin levels were significantly reduced in leaf explants incubated in liquid media (Fig. 5C, D), suggesting that drought stress-induced anthocyanin accumulation also occurs in leaf explants during tissue culture. Next, we examined whether anthocyanin levels affect root organogenesis in leaf explants. Rooting rates of leaf explants on agar and liquid media did not show any significant differences until 14 DAC (Fig. 5E), suggesting that anthocyanin levels do not affect root organogenesis.

**Fig. 6:**
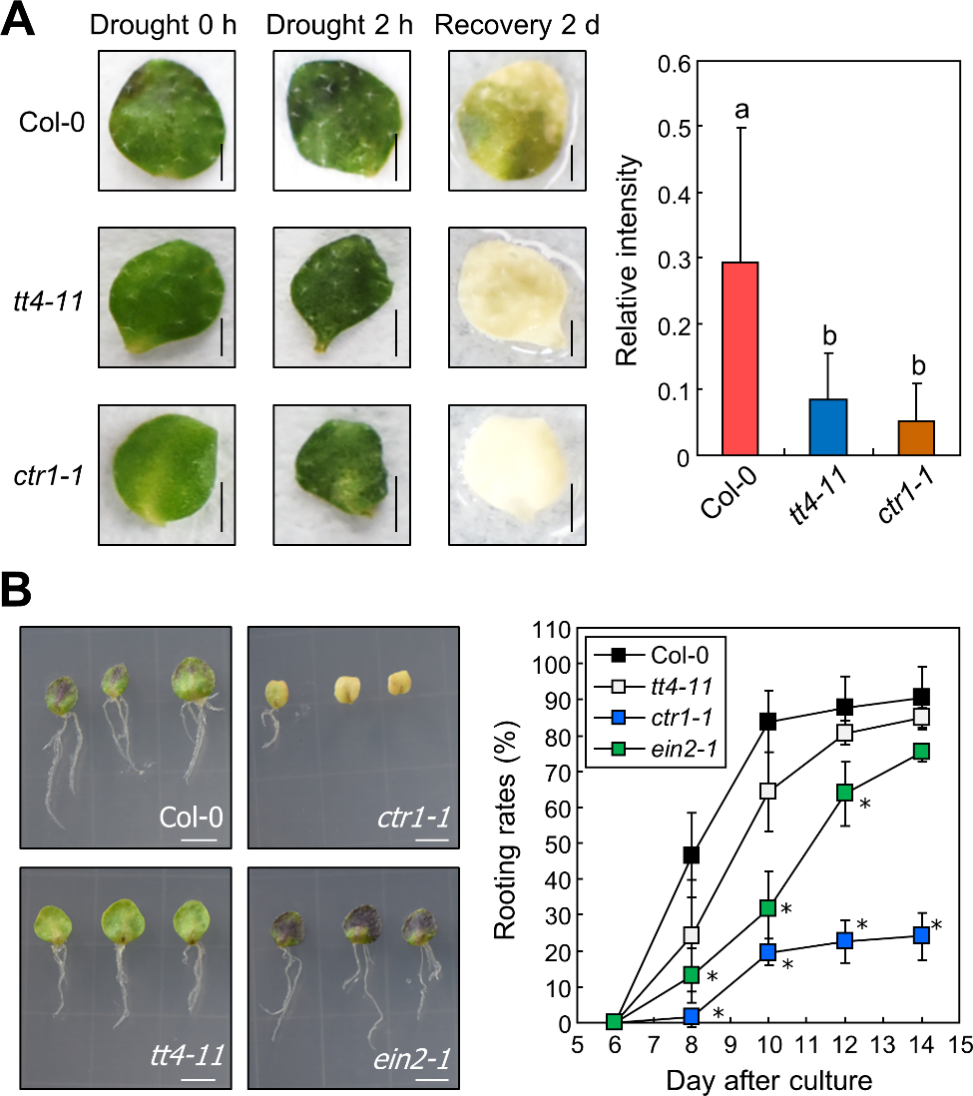
Anthocyanins improve drought resistance of leaf explants. (**A**) Leaf explants of the 9-day-old Col-0, *tt4-11*, and *ctr1-1* seedlings were incubated on B5-agar plates for 5 days. Explants were placed on filter paper for 2 h to induce drought stress and then 2 ml of distilled water was supplemented for recovery. Relative intensity of leaf color was measured at 2 days after recovery using ImageJ software. Three biological replicates were averaged. Letters indicate groups that are statistically significantly different from each other (*P* < 0.05, Tukey’s test). Each replicate contains 12–14 explants. Size markers indicate 0.25 cm. Whiskers indicate + SD. (**B**) Rooting rates of ethylene signaling and anthocyanin biosynthesis mutants. Leaf explants of the 9-day-old Col-0, *tt4-11*, *ctr1-1*, and *ein2-1* seedlings were incubated on B5-agar plates up to 14 days. Three biological replicates were averaged and statistically analyzed using Student’s *t*-test (*P < 0.05; difference from Col-0). Each replicate contains 20–25 explants. Size markers indicate 0.5 cm. Whiskers indicate ± SD

Flavonoids, including anthocyanins, confer drought tolerance to plants [43, 44]. As our results showed that anthocyanin accumulation in leaf explants is not related to root organogenesis, we hypothesized that accumulated anthocyanins affect drought resistance. We thus examined the survival of leaf explants under drought stress using anthocyanin-deficient *tt4-11* and *ctr1-1* mutants. Excised leaves were incubated on B5-agar medium for 5 days and subjected to drought stress on dry filter paper. Intensity of green color was measured as an indicator of chlorophyll content and resistance to drought-induced cell death after 2 days of recovery. Although anthocyanin-deficient *tt4-11* and *ctr1-1* lost their green color, Col-0 explants showed a relatively higher intensity of green color (Fig. 6A), suggesting that anthocyanins might be required for drought resistance in leaf explants. However, we cannot entirely rule out the possibility that reduced drought tolerance in *tt4-11* and *ctr1-1* mutants is caused by other functions of TT4 and CTR1, which are not related to anthocyanin biosynthesis.

Next, we compared the rooting rates of leaf explants between anthocyanin-deficient *tt4-11* and *ctr1-1* mutants and anthocyanin over-accumulating *ein2-1* mutant. Similar to our observation that anthocyanin content did not affect root regeneration (Fig. 5C to E), *tt4-11* and Col-0 were found to exhibit similar rooting rates until 14 DAC (Fig. 6B). However, the *ein2-1* exhibited slow rooting rate compared to Col-0, but the final rooting rates at 14 DAC were similar in *ein2-1* and Col-0. Meanwhile, *ctr1-1* exhibited significantly reduced rooting rate compared to Col-0 at all time points in our experiments. Because altered anthocyanin levels did not affect root regeneration, these results might be due to the altered ethylene signaling in these mutants [45, 46].

### ROS induce lignin accumulation at the wound site

Anthocyanins and lignins share upstream biosynthetic pathways starting with phenylalanine [47]. As lignins act as barriers to block pathogen infections [48], we examined whether lignins accumulate near the wound site in leaf explants using phloroglucinol-HCl, which stains the 4-O-linked hydroxycinnamyl aldehyde structures of lignins [49, 50]. Phloroglucinol staining revealed primary lignin deposition at the wound site, where anthocyanins did not accumulate, 1 DAC (Fig. 1A and S1A). Lignin signals increased during incubation until 5 DAC. However, similar pink-red colors were also observed at SDW where anthocyanins accumulated, possibly because the low pH of phloroglucinol-HCl turns anthocyanin color to red [51]. To distinguish between anthocyanins and lignins, we used anthocyanin-deficient *tt4-11* mutant as a control. Although the pink-red color at SDW after phloroglucinol staining was diminished by *tt4-11* mutation, staining signals at the wound site were still observed in both Col-0 and *tt4-11* explants 5 DAC (Fig. S1B). These results indicate that, unlike anthocyanins, lignins are deposited at the wound site of leaf explants.

Next, we performed phloroglucinol staining using *ein2-1* mutants to determine whether ethylene signals affect lignin deposition. We found that lignin signals at the wound site were not affected by *ein2-1* mutation (Fig. S1C), suggesting that ethylene is not mainly involved in lignin deposition in leaf explants. To further identify the upstream regulators of lignin deposition at the wound site, we analyzed NADPH oxidase RESPIRATORY BURST OXIDASE HOMOLOG (RBOH)-deficient mutants as RBOHD- and RBOHF-produced ROS control lignin deposition in flowers [52]. Although the *rbohD* mutation did not affect lignin deposition, *rbohDF* double mutations diminished the lignin signals at the wound site 5 DAC (Fig. S1D, E). These results indicate that RBOHD- and RRBOHF-mediated ROS production after wounding induces lignin deposition at the wound site independent of anthocyanin and ethylene signals.

## Discussion

In this study, we found that ethylene signals regulate anthocyanin accumulation and expression of pathogen resistance genes in leaf explants. In our signaling scheme, wounding may induce ethylene biosynthesis mainly at SNW through upregulation of *ACS* expressions (Fig. S2). Ethylene then inhibits anthocyanin accumulation and induces expression of *PDF1.2*, *PR3*, and *PR4* at this region. In contrast, relatively weak ethylene signals allow anthocyanin accumulation at SDW. As induction of *PR* and *PDF* expressions confers pathogen resistance [53–55] and anthocyanin accumulation improves drought resistance (Fig. 5) [43], our observations indicate that wounding triggers these responses via ethylene signals for the survival of leaf explants under both abiotic and biotic stresses.

Wounding is unavoidable to generate explants for plant tissue culture. However, pathogens can easily invade the plant tissues via the wounded parts [56]. Therefore, in many plant species, defense responses are activated by wounding. In Arabidopsis, wounding promotes camalexin production for defense against *B. cinerea* [57]. In tomato, accumulation of feruloyltyramine and p-coumaroyltyramine, which confer resistance against *Xanthomonas campestris*, is increased in leaves after wounding [58–60]. In this study, we found that wound-induced defense responses are also activated in leaf explants during tissue culture. Expression of *PR3*, *PR4*, and *PDF1.2* genes, which are involved in ethylene-mediated defense responses [18, 19, 22], increased at SNW in an EIN2-dependent manner (Figs. 3 and 4). Therefore, our observations suggest that ethylene locally induces expression of these genes near the wound site, where pathogen invasion is suspected.

During tissue culture, leaf explants face drought stress despite being cultured on agar medium because of the absence of roots. Therefore, leaf explants activate auxin signals for root organogenesis [61]. However, the explants may need to induce drought stress resistance responses prior to root development. As ROS production is critical for drought-induced cell death [62], ROS-scavenging processes would be essential in leaf explants. We found that leaf explants accumulate anthocyanins particularly at SDW (Fig. 1). Anthocyanins act as antioxidants that scavenge ROS and enhance tolerance to drought stress [39, 43]. Indeed, anthocyanin over-accumulating plants exhibit high survival rates, whereas anthocyanin-deficient plants exhibit low survival rates under drought and ROS stress conditions [39, 43]. Consistent with previous reports, anthocyanin-deficient *tt4-11* and *ctr1-1* explants exhibited significantly low survival rates under drought conditions in our data (Fig. 6A). These results suggest that antioxidants accumulate at non-wounded sites, where ethylene signals are relatively weak, to confer drought tolerance to leaf explants.

Notably, lignin signals were not observed in *rbohDF* double mutants (Fig. S1E), indicating the important roles of RBOHD and RBOHF in lignin accumulation. As RBOHs are NADPH oxidases that produce ROS in plant cells [63, 64], ROS would be required for lignin biosynthesis in leaf explants. Indeed, ROS induce lignin biosynthesis in roots and flowers and accumulate at the wound site in leaf explants [32, 50, 52]. Here, anthocyanins accumulated very slowly near the wound site (Fig. 1A, B), indicating that ROS-mediated lignin deposition may be supported by ethylene-mediated inhibition of anthocyanin biosynthesis at the wound site. Lignins are physical barriers that restrict pathogen invasion in plant cells [48]; therefore, lignin deposition at the wound site may be another defense mechanism to minimize pathogen infection via disrupted tissues in leaf explants.

## Methods

### Plant materials and growth conditions

*Arabidopsis thaliana* ecotype Columbia (Col-0) was used in this study. The *coi1-21* (N68754), *rbohD* (N9555), *rbohDF* (N9558), and *tt4-11* (N2105573) seeds were obtained from the Nottingham Arabidopsis Stock Centre (NASC, Nottingham, UK). The *ctr1-1* [34], and *ein2-1* [65] seeds were a gift from Dr. Young-Joon Park.

Seeds were surface-sterilized in 75% (v/v) ethanol with 0.03% (v/v) Triton X-100, and then washed with 70% (v/v) ethanol two times. After 3 days of stratification at 4 °C, seeds were transferred to growth room set at 24 °C with 40–50% humidity under long-day conditions. The seedlings were grown on 1/2 Murashige and Skoog (MS)-agar plates containing 0.05% (w/v) of MES and 0.7% (w/v) of plant agar with pH 5.7. The plates were exposed to white light with an intensity of 100 µmol m^− 2^ s^− 1^ using fluorescent FL40EX-D tubes (Focus, Bucheon, Korea).

### Measurement of anthocyanin content

Seedlings were grown on MS-agar plates for 9 days. Leaves or seedlings excised at the indicated site in figures. Mainly, blade-petiole junctions were excised for leaf explants. For leaf explants, first rosette leaves were used. The explants were incubated on Gamborg B5-agar plates containing 0.05% (w/v) of MES and 0.7% (w/v) of plant agar with pH 5.7 for the indicated time periods in figures. The explants were exposed to same light intensity of growth conditions. The plant materials were placed on MS-agar plates and photographed using a Nikon D5600 digital camera and STEMI 2000-C stereo microscope (Carl Zeiss). About 1.2 mm from the wound site was defined as SNW and the remaining part was defined as SDW. For measuring anthocyanin content, SNW and SDW were separately harvested and incubated in 300 µl of methanol containing 1% (v/v) HCl for 16 h at 4 °C. After the extraction, 200 µl of distilled water and 200 µl of chloroform were added for separating chlorophylls from anthocyanins. The mixture was centrifuged at 16,000 X g for 10 min at 4 °C. The supernatant was transferred to a new microcentrifuge tube and 500 µl of distilled water was added. Absorbance was measured at 535 nm (A535) using a spectrophotometer (Beckman Coulter, Brea, CA, United States). Anthocyanin contents were calculated as follows:

Anthocyanin contents = A535 / fresh weight (mg)

### Phloroglucinol staining

Seedlings were grown on MS-agar plates for 9 days and first rosette leaves were detached. The leaf explants were incubated on Gamborg B5-agar plates for the indicated time periods in figures. The explants were immersed in phloroglucinol staining solution containing 1% (w/v) of phloroglucinol in 50% (v/v) of ethanol and 50% (v/v) HCl. After inverting several times, the plant materials were mounted on MS-agar-plates and photographed using a STEMI 2000-C stereo microscope (Carl Zeiss).

### ***De novo*** root organogenesis of leaf explants

Seedlings were grown on MS-agar plates for 9 days and first rosette leaves were detached. The explants were incubated on Gamborg B5-agar plates or floated on B5 liquid for up to 14 days to induce *de novo* root organogenesis. The rooting rate of explants was measured at intervals of two days starting at 6^th^ day after culture.

### Drought stress assays of leaf explants

Seedlings were grown on MS-agar plates for 9 days and first rosette leaves were detached. The leaf explants were incubated on Gamborg B5-agar plates for 5 days. To induce drought stress, the explants were placed on filter paper at room temperature for 2 h. For recovery, explants were incubated in growth room for 2 days after supplying distilled water. The plant materials were photographed using a Nikon D5600 digital camera at the indicated time periods in figures. The ImageJ software was used to measure the leaf color intensity.

### RNA extraction and gene expression analysis by quantitative PCR

The leaf explants from the seedling grown for 9 days on MS-agar plates were incubated on Gamborg B5-agar plates for the indicated time periods in figures. The SNW and SDW were harvested separately. Total RNA was extracted from the plant materials using Trizol (Thermo Fisher Scientific) according to the manufacturer’s recommendations. First-strand complementary DNA synthesis was performed using AccuPower CycleScript RT PreMix (Bioneer) according to the manufacturer’s protocol. Quantitative PCR (qPCR) was performed using TOPreal qPCR 2X PreMIX (SYBR Green with low 5-carboxy-x-rhodamine, Enzynomics) in CFX Connect PCR device (Bio-Rad). The comparative ΔΔC_T_ method was used to calculate relative quantities of each amplified PCR product. The threshold cycle (C_T_) was determined using the Bio-Rad CFX Manager software with default parameters. Primers used for qPCR were listed in Table S1. The gene expression was normalized using *UBQ10* as a reference gene.

### Statistical analysis

To determine statistically significant differences, one-way analysis of variance (ANOVA) with *post hoc* Tukey’s test and Student’s *t*-test were performed using Rstudio and Excel software, respectively.

## Electronic supplementary material

Below is the link to the electronic supplementary material.

Supplementary Material 1

## Data Availability

All generated or analyzed data were included in this article. The raw datasets obtained during the current study are available from the corresponding author on reasonable request.
