# Supplementary material for: Ethylene signals modulate the survival of Arabidopsis leaf explants
Source: BMC Plant Biol. 2023 May 26;23:281. doi: 10.1186/s12870-023-04299-4 (PMC10214724; doi:10.1186/s12870-023-04299-4)
Supplement: Supplementary file 1 — Supplementary Material 1 [file 12870_2023_4299_MOESM1_ESM.pdf]

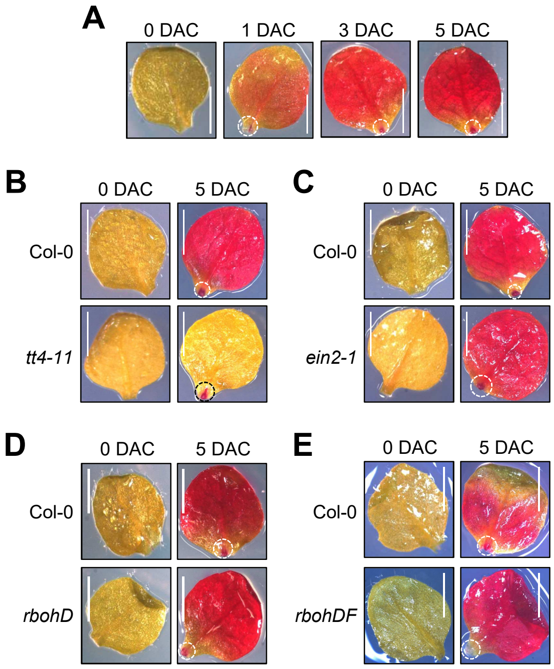

Fig. S1 Accumulation of lignins at the wound site in leaf explants.
Leaf explants of the 9-day-old Col-0 (A-E), *tt4-11* (B), *ein2-1* (C), *rbohD* (D), and *rbohDF* (E) seedlings were incubated on B5-agar plates for the indicated time periods. Representative images are displayed. Size markers indicate 0.25 cm. White and black dotted circles indicate wound sites.

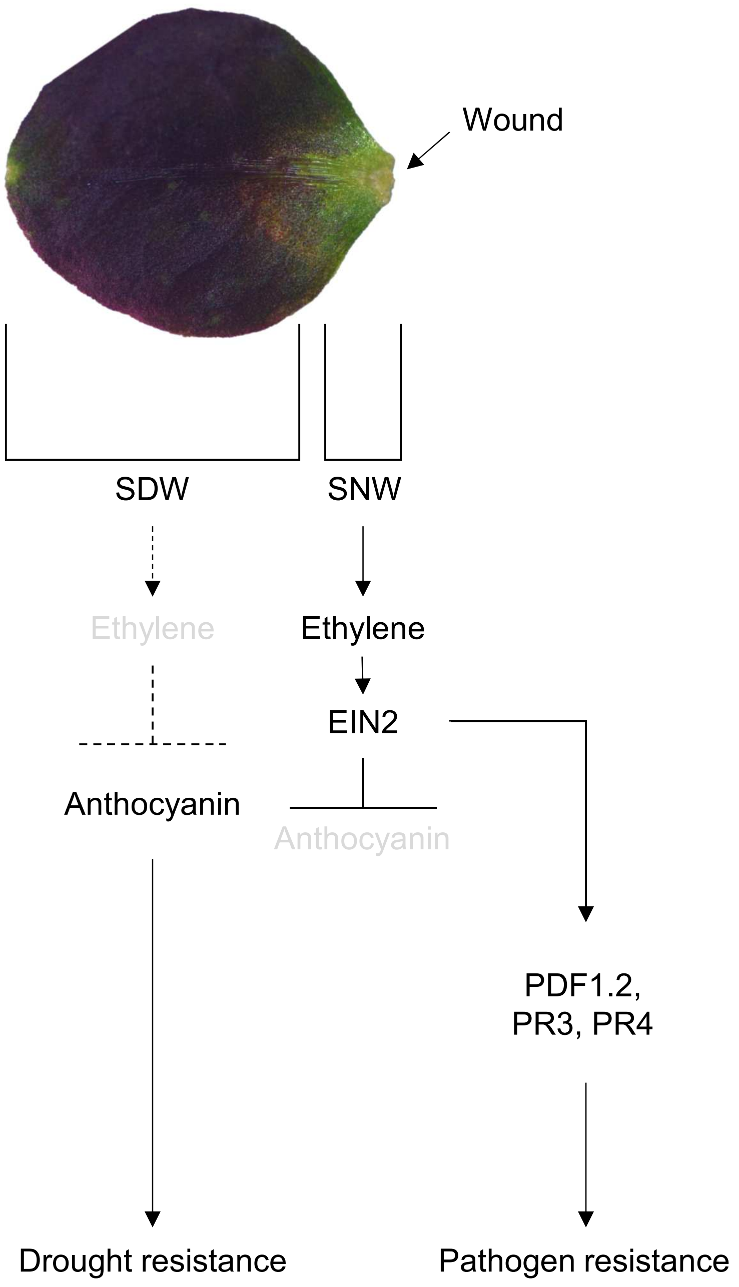

Fig. S2 Proposed working model for the role of ethylene in leaf explants.
SDW and SNW indicate the site distant from the wound and the site near the wound, respectively. Dotted lines indicate weak signal. In leaf explants, ethylene might be mainly produced at SNW. Increased ethylene signals inhibit anthocyanin accumulation through EIN2, while they up-regulate expression of *PDF1.2, PR3,* and *PR4,* which are related to pathogen resistance. At SDW, relatively weak ethylene signals allow accumulation of anthocyanins, which improves drought resistance.

Primers	Sequences	Usage
UBQ10-F	CGACCCTTCACTTGGTGTTG	RT-qPCR
UBQ10-R	TTTCCAGCGAAGATGAGACG	"
ACS2-F	CAGTTTTGTCGAAGATAGCG	"
ACS2-R	GATTCTCTGCAAGACCCATT	"
ACS6-F	GAAAATGTCGAGTTTCGGTC	"
ACS6-R	TAAACCATCTAAACCGGTGG	"
ACS7-F	TAACTTTCATTCTCGCCGAT	"
ACS7-R	GGGGTTATCTGGAAATGGTT	"
ACS8-F	GCTAACGAGACTCTCATGTT	"
ACS8-R	GAAACCGTTTGCACTCTTAC	"
PAL1-F	ACACTGTCTCTCAAGTGGCG	"
PAL1-R	GAATCAACGGGTACGTTGCG	"
PAL2-F	GATCACGCTTTGTCCAACGG	"
PAL2-R	AATCCGGTTAGGAATCGGCG	"
PAL3-F	GGGATATTCGCTACCGGCAA	"
PAL3-R	TAATGGTGCCTCGGAGAGGA	"
TT8-F	AAGACGGCGGTTCAATCTGT	"
TT8-R	CTGAGCTGTTGGCTCCTCTC	"
PR1-F	ACGGGGAAAACTTAGCCTGG	"
PR1-R	TTGGCACATCCGAGTCTCAC	"
PR2-F	TCGAACCAGTGATAGGTTTC	"
PR2-R	AGTACCCTGGATCGTTATCA	"
PR3-F	TCAAAGCCGCGATTTGGTTC	"
PR3-R	CACGTCCACACTCCAATCCA	"
PR4-F	ATGCTGATAAGCCGTACGCA	"
PR4-R	CAAATCCAAGCCTCCGTTGC	"
PR5-F	AATGTCAAGCTGGGGATAAG	"
PR5-R	ATTAAACCTCTCACAGGCAC	"
PDF1.2-F	GCTTTCGACGCACCG	"
PDF1.2-R	TAGTTGCATGATCCATGTTTG	"
PAP1-F	TGCTGGAAGATTACCTGGTCG	"
PAP1-R	TGCCGGTGTTGTAGGAATGG	"
DFR-F	GTGGGATTTCGCCGAAGAGA	"
DFR-R	GCCTCGTTCCGAGTGATAGG	"
ANS-F	TGGTTGCGGTTGAAAGAGTTG	"
ANS-R	TTGTGGGAACTTGAGGACCG	"
UF3GT-F	GACAAACTCCGACGTCCCTT	"
UF3GT-R	CCGGTATCCAATGGGCAGAA	"

Table S1 Primers used in this study.
The primers used were designed using the Primer Blast (https://www.ncbi.nlm.nih.gov/tools/primer-blast). F, forward primer; R, reverse primer.
